# Resolutions of Respect to Dr. W. D. Tenison

**Published:** 1900-05

**Authors:** B. C. Nash

**Affiliations:** Secretary


### Obituary.

### RESOLUTIONS OF RESPECT TO DR. W. D. TENISON.

At a meeting of the First District Dental Society of the State of New York, March 13, 1900, the following resolutions were presented by Dr. John I. Hart, and adopted:

Whereas, Dr. William Deane Tenison has been removed by death, it becomes our painful duty to take notice of his demise; therefore be it

*Resolved*, As the sense of this society, that in the death of Dr. Tenison the profession has lost a distinguished member. As a man Dr. Tenison was genial and affable; as a dentist he was skilful and conscientious. Entering upon the study of dentistry at an early age when all was crude, he wrought his way to success and high position by zeal and industry. We shall miss Dr. Tenison from our ranks. Let us therefore linger for a moment to pay his memory this tribute of respect.

*Resolved*, That a copy of these resolutions be sent to his family and the dental journals.

B. C. Nash,
*Secretary.*

